# Supplementary figures and images for: A simple dissection method for the isolation of mouse trabecular meshwork cells
Source: PLoS One. 2023 Dec 21;18(12):e0296124. doi: 10.1371/journal.pone.0296124 (PMC10734917; doi:10.1371/journal.pone.0296124)

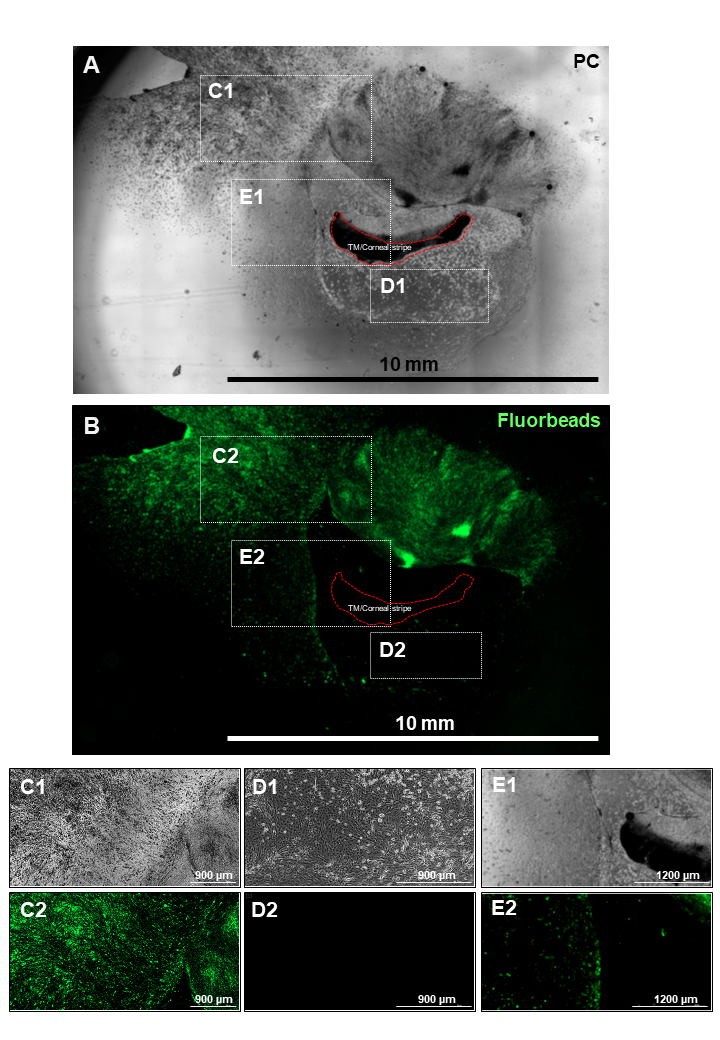

Supplement: S1 Fig — The addition of fluorobeads for 48 h and subsequent washing steps reveals that only one cell layer exhibits phagocytotic properties. (TIF) [file pone.0296124.s003.tif]
