# Supplementary material for: A simple dissection method for the isolation of mouse trabecular meshwork cells
Source: PLoS One. 2023 Dec 21;18(12):e0296124. doi: 10.1371/journal.pone.0296124 (PMC10734917; doi:10.1371/journal.pone.0296124)
Supplement: Attachment — Submitted filename: Review Comments_PONE-D-23-21644.pdf [file pone.0296124.s005.pdf]

Review Comments

A simple and animal-reducing dissection method for the isolation of mouse trabecular meshwork cells

Maximilian Binter, Fridolin Langer, Xiaonan Hu, Migle Lindziute, Carsten Framme, Jan Tode, Heiko Fuchs*

Summary

In present study, authors showed dissection method to isolate mouse trabecular meshwork (TM) cells from C57BL/6J mice. Isolated cells were characterized with (i) immunocytochemistry (ICC) for the known markers Collagen IV, Fibronectin I, Vimentin (ii) myocilin (Myoc) induction by dexamethasone (DEX) with ICC & qPCR technique (iii) increased Cross-Linked Actin Networks (CLANs) upon exposure to TGFB2 via ICC, and (iv) phagocytic properties using fluorescent microbeads. Such analysis clearly demonstrated successful isolation of TM cells.

Overall, study clearly demonstrated successful isolation of TM cells from wild type C57BL/6J mice but lacks the evidence to support their claim that their method is simple, easily re-producible, inexpensive, animal reducing and high yield! **I recommend it for major revision in this regard.**

Major comments:

- Authors claimed that their method is simple, easily re-producible, inexpensive, animal reducing and high yield! However, data do not support those claims and below are some reasons to conclude it:
  1) Dissection method is not very different from what is described by Tamm *et. al.* 1999, so it's not justified that the method used here is anyway simpler or better! It's only the assay demonstrating phagocytic properties of isolated TM cells which wasn't available in earlier time.
     So, it is possible to do the same assay with TM cells isolated by Tamm et. al. if they have frozen stock available, then compare! This can provide some conclusive evidence.

2) There were 12 animals (24 eyes) used, and they were wild-type C57BL/6J mice. No quantification data showing the total yield of TM cells obtained. It is important to prove the claim of "high-yield" method. Also, missing control to even compare the qualitative properties!
3) As claimed (and potentially true) that this method can be applied to isolate TM cells from other mouse models; authors should demonstrate this using at least with one other mouse model. This can also demonstrate efficiency of the method as well as total yield comparison, and Animal reduction aspect too!

- **TGFB2 exposure and CLAN analysis:** Not clear if the % of CLAN positive cells are given as per 10 coverslips or 10 x 5 = 50 regions observed or per 1274 analyzed cells? What % of the total cells the 1274 cells represent?

Minor comments:

- Scale bars need to be defined for figures 4 & 5.
